# XCHD Inhibits C6 Cell Growth Primarily via the p53/Caspase Pathway

**DOI:** 10.1155/2020/7973639

**Published:** 2020-09-22

**Authors:** Huiling Yu, Qinnuan Sun, Fen Yun, Xiaoyan Xu, Feng Ding, Shirong Li, Pengwei Zhao

**Affiliations:** School of Basic Medical Science, Inner Mongolia Medical University, Hohhot, China

## Abstract

The effects of XCHD on the proliferation of C6 cells and on factors associated with the microRNA-34a (miR-34a)/p53/caspase-3 signaling pathway *in vitro* were investigated. *Methods*. XCHD was purchased too much to complete the study. CCK-8 assay was used to measure the XCHD concentration, and qPCR was used to quantify miR-34a expression at the mRNA level. Apoptosis was assessed using TUNEL. Western blots were used to determine the p53, caspase-3, caspase-8, and Bcl-2 expression levels. *Results*. The optimal XCHD concentration and time effect for C6 cells were observed after 36 h of exposure to a concentration of 100 *µ*g/ml XCHD. miR-34a expression increased 8 and 12 h after the addition of XCHD. The presence of XCHD decreased Bcl-2 expression but increased p53, cleaved caspase-3, Bax, and caspase-8 expression. When p53 was inhibited, miR-34a expression was unaffected by the addition of XCHD, Bcl-2 expression was low, and cleaved caspase-3, Bax, and caspase-8 expression increased. The inhibition of p53 promoted C6 cell growth when compared with C6 cells exposed to XCHD and with no inhibition of p53. *Conclusions*. XCHD inhibits C6 cell growth which was influenced by the p53/caspase pathway.

## 1. Introduction

Glioma cells are the most common type of intracranial primary tumor cells, and they exhibit invasive growth and possess a high postoperative recurrence [1, 2]. Glioblastomas pose a serious threat to the lives and health of patients. Many patients do not recover from this type of cancer even after undergoing operations, radiotherapy, and chemotherapy [3]; their median survival time is approximately 14.6 months [4]. Treating glioblastomas is difficult because a very small fraction of small cell subsets (1%) cannot be killed after being treated with radiotherapy and chemotherapy [5–7]. Therefore, the investigation of novel potential therapeutic agents, such as those used in traditional Chinese medicine (TCM), and the molecular mechanisms underlying their cytotoxic effect via clinical investigations is necessary [3, 4, 8].

TCM is widely used in China and is becoming increasingly prevalent in Europe and America. Several complex formulas and crude materials used in Chinese medicines, including Xiaochaihu decoction (XCHD), have demonstrated measurable activity against viral hepatitis in the central nervous system [9–11]. XCHD is a classic formulation, which is described in a “Treatise on Cold Pathogenic and Miscellaneous Diseases” as having anti-inflammatory effects and providing gastric protection and regulation of the immune system [12–14]. XCHD reduces the number of preneoplastic cells, inhibiting the development of liver tumors [15–17]. However, few studies have focused on the efficacy of XCHD in terms of its effects on primary tumors of the central nervous system such as glioma.

The cellular response to gliomas has been widely studied; however, the role of microRNA (miRNA/miR) in gliomas remains largely unknown [18, 19]. The functions of many miRNAs are associated with gliomas [20]. Because of the numerous functions of miRNA, studies involving the miRNA profile associated with gliomas are necessary [21].

p53, a transcription factor that stops the cell cycle and induces a proapoptotic effect via the modulation of multiple target genes, is another well-known tumor suppressor in cancer research [22, 23]. In murine brains, p53 deficiency plays a central role in driving gliomagenesis [24]. Moreover, p53 deficiencies contribute to neuronal death after transient cerebral ischemic injury, while delayed treatment with p53 inhibitor could facilitate endogenous neurogenesis and therefore improve functional recovery [25]. Several p53-regulated miRNAs have been implicated, including the miR-17-92 cluster, miR-145, and let-7 [23]. Importantly, p53 regulates tumor cell recognition via the p53-regulated miRNA miR-34a [23].

To elucidate the possible molecular mechanism of using XCHD to treat glioma, a serum pharmacological method was used in the present study. The effects of XCHD on the proliferation of C6 cells and on factors associated with the miR-34a/p53/caspase-3 signaling pathway *in vitro* were investigated.

## 2. Materials and Methods

XCHD was purchased from the Chinese National Institute (Beijing, China). A decoction of XCHD (chaihu, huangqin, renshen, gancao, banxia, shengjiang, and dazao) was prepared from a mixture of chopped crude herbs, extracted in distilled water at 100°C; this mixture was then concentrated to a final concentration of 100 crude drug gram per 100 milliliters. And primary antibodies were purchased from Abcam. miRNA expression assay kits were purchased from Tiangen, and p53 inhibitor pifithrin-*α* was purchased from Sigma.

### 2.1. Cell Culture

C6 glioma cells (Chinese Academy of Sciences, China) were cultured at 37°C in a humidified atmosphere containing 5% CO_2_.

### 2.2. Cell Proliferation Assay

A Cell Counting Kit-8 (CCK-8) assay was used to measure cell viability and proliferation. Cells were seeded in 96-well plates with different concentrations of XCHD (0, 10, 50, 100, and 200 *µ*g/ml) and were incubated for different periods of time (12, 24, 36, and 48 h).

### 2.3. Western Blotting

Cell protein samples were separated via 12% sodium dodecyl sulfate-polyacrylamide gel electrophoresis (SDS-PAGE) and transferred to polyvinylidene fluoride (PVDF) membranes. The membranes were blocked in 5% nonfat milk diluted with Tris-buffered saline/Tween 20 (TBST) at room temperature for 1 h and incubated overnight at 4°C with primary antibodies. The membranes were then washed three times and developed using enhanced chemiluminescence (ECL) reagents (Pierce, Rockford, USA).

### 2.4. Flow Cytometric Analysis

Cells (1 × 10^6^) were washed with phosphate-buffered saline (PBS) and then labeled using Annexin V and propidium iodide (PI). Apoptotic rates were measured using flow cytometry and FlowJo software. The percentage of apoptotic cells was measured by applying the criteria of Annexin V positivity and PI negativity, while the percentage of late apoptotic cells was determined by applying the criteria of Annexin V positivity and PI positivity.

### 2.5. Analysis of Quantitative Reverse Transcriptase Polymerase Chain Reaction (qRT-PCR) Results

Total cellular RNA was obtained using an RNA extraction kit, and complementary DNA (cDNA) was synthesized from 1 *μ*g of total RNA. RT primers and a TaqMan miRNA assay kit (Applied Biosystems, USA) were used to perform reverse transcription and PCR according to the manufacturer's instructions.

### 2.6. Statistical Analysis

One-way analysis of variance (ANOVA) was performed using GraphPad Prism version 5 software. The data are presented as the mean ± standard deviation (SD) *P* values less than 0.05 were considered significant.

## 3. Results

### 3.1. Effects of XCHD on C6 Glioma Cell Growth and XCHD Concentration

A 3-[4,5-dimethylthiazol-2-yl]-2,5 diphenyl tetrazolium bromide (MTT) assay was used to assess cell growth and viability following treatment of C6 cells with different concentrations of XCHD and determine the appropriate treatment duration to achieve the desired level of cellular growth.

**Figure 1:**
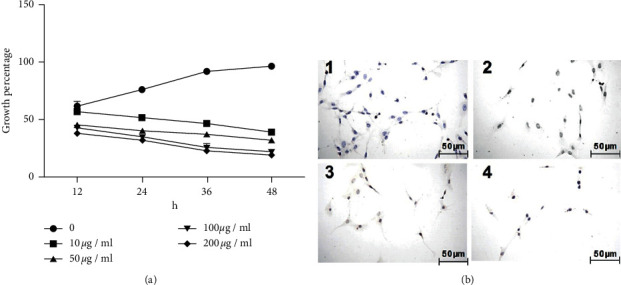
XCHD effect on the growth of C6 cells. (a) Growth percentage of different concentrations (0, 10 *μ*g/ml, 50 *μ*g/ml, 100 *μ*g/ml, and 200 *μ*g/ml) XCHD effected on the C6 cells at 12 h, 24 h, 36 h, and 48 h. (b) TUNEL detected the C6 cell apoptosis status effected by XCHD with 100 *μ*g/ml at 12 h, 24 h, 36 h, and 48 h. A: 12 h, B: 24 h, C: 36 h, and D: 48 h.

XCHD was added to the growth medium to achieve final XCHD concentrations of 0, 10, 50, 100, and 200 *µ*g/ml. The growth percentages obtained for each XCHD concentration were assessed at 12, 24, 36, and 48 h (Figure 1(a)). The results showed the optimal concentration and XCHD treatment duration for the attenuated growth of C6 cells were 100 *µ*g/ml XCHD and 36 h, respectively.

To further examine the effects of XCHD on C6 cells, terminal deoxyribonucleotidyl transferase-mediated biotin-16-dUTP nick-end labeling (TUNEL) was used to study the effects of 100 *µ*g/ml XCHD on C6 cells at 12, 24, 36, and 48 h. The results (Figure 1(b)) showed the cell concentrations at 36 and 48 h were very low.

### 3.2. Effects of XCHD on miR-34a Expression in C6 Cells

**Figure 2:**
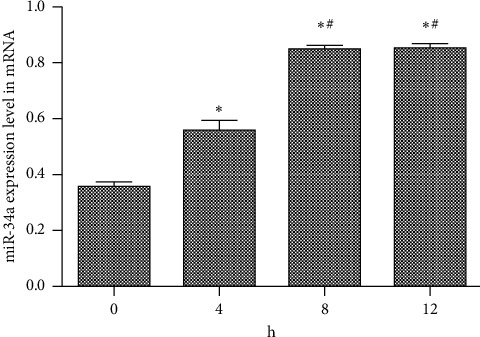
Relative miR-34a expression levels effected by XCHD. ^*∗*^*p* < 0.05 versus 0. ^#^*p* < 0.05 versus 4 h.

Quantitative PCR (qPCR) was used to determine miR-34a expression at the mRNA level after the addition of XCHD. The results (Figure 2) showed miR-34a expression levels were higher at 8 and 12 h than those at 0 and 4 h (*P* < 0.05). Furthermore, at 4 h, the miR-34a expression level was higher than that at 0 h (*P* < 0.05).

### 3.3. XCHD Attenuated C6 Growth via the p53/Caspase Pathway

**Figure 3:**
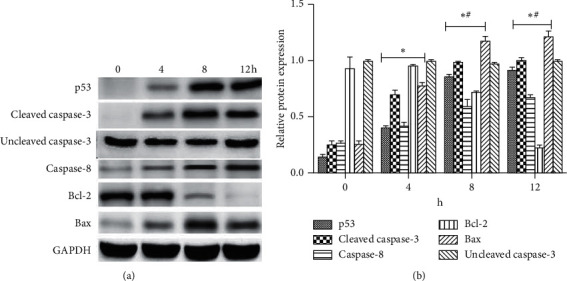
p53, cleaved caspase-3, caspase-8, Bcl-2, and Bax protein expression effected by XCHD. (a) The expression of p53, cleaved caspase-3, caspase-8, Bcl-2, and Bax proteins. (b) Relative protein expression (protein/GAPDH) of p53, cleaved caspase-3, caspase-8, Bcl-2, and Bax based on the western blot.

**Figure 4:**
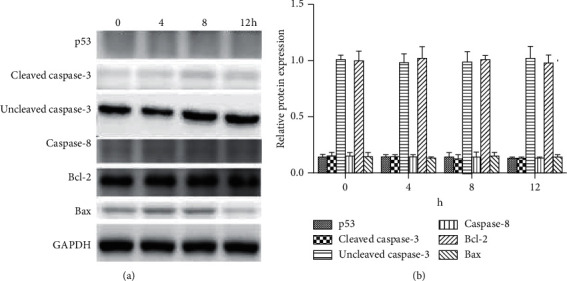
Influence of C6 cell after p53 inhibition. (a) The expression of p53, cleaved caspase-3, caspase-8, Bcl-2, and Bax proteins. (b) Relative protein expression (protein/GAPDH) of p53, cleaved caspase-3, caspase-8, Bcl-2, and Bax based on the western blot.

**Figure 5:**
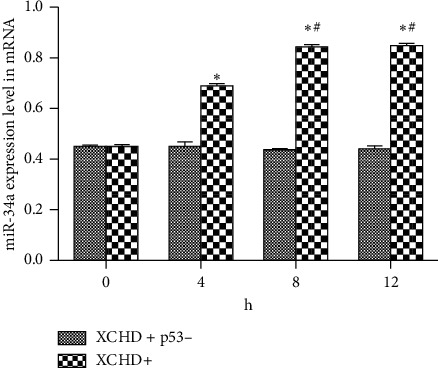
Relative miR-34a expression levels. Influence of C6 cell after p53 inhibition. ^*∗*^*p* < 0.05 versus 0. ^#^*p* < 0.05 versus 4 h.

**Figure 6:**
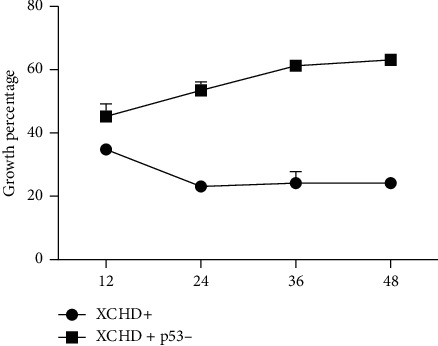
Growth percentage effected on the C6 at 12 h, 24 h, 36 h, and 48 h.

To determine the signaling pathways mediating the attenuating effect of XCHD on C6 cell growth, the p53, cleaved caspase-3, caspase-8, Bcl-2, and Bax protein levels were quantified after 0, 4, 8, and 12 h (Figure 3(a)) of exposure to 100 *µ*g/ml XCHD. XCHD exposure decreased the Bcl-2 protein level; however, the p53, cleaved caspase-3, caspase-8, and Bax protein levels increased after XCHD exposure (Figure 3(b)). These results showed p53/caspase signaling plays a key role in the inhibitory effect of XCHD on C6 cell growth. To verify these results, p53 inhibitor was used to silence p53 expression in addition to XCHD. Bcl-2 expression was low during the entire experimental period in C6 cells exposed to both the p53 inhibitor (120 nM) and XCHD, but the Blc-2 expression level was higher than that of the conditions that did not use p53 inhibitor. In addition, the cleaved caspase-3, caspase-8, and Bax protein levels were higher than those obtained with XCHD alone (Figure 4). miR-34a expression was lower in C6 cells exposed to both XCHD and the inhibitor of p53 expression (Figure 5). The inhibition of p53 expression promoted C6 growth when compared with the effect of XCHD exposure only (Figure 6).

## 4. Discussion

Brain glioma tumors are often lethal to humans [26, 27]. Numerous studies have demonstrated XCHD and its active components exhibit notable therapeutic effects in cancer cells when using XCHD as a TCM [28, 29]. In this study, XCHD inhibited the growth of C6 cells via the p53/caspase pathway.

XCHD, derived from a classic TCM described in the “Treatise on Febrile Diseases,” has exhibited positive effects in the treatment of chronic hepatitis B (CHB) in China and is a classic treatment method for promoting bile flow [30]. XCHD can be anticancer [28]. The results of this study showed XCHD inhibits the growth of C6 cells.

XCHD is composed of Chinese thorowax root, *Scutellaria baicalensis*, *Pinellia ternata*, ginger, ginseng, *Ziziphi jujubae*, liquorice root, *Phyllanthus urinaria*, largehead atractylodes rhizome, *Poria cocos*, white peony root, and *Fructus schisandrae*. XCHD inhibits the growth of liver cancer cells via the JAK2/STAT3 pathway [31]. The results of this study demonstrated XCHD attenuated the growth of C6 cells via activation of the miR-34a/p53 pathway. The addition of XCHD promoted the expression of p53 and increased miR-34a. In addition, miR-34a expression levels were correlated with the rate of apoptosis in C6 cells, in which tumor suppressor p53 activated miR-34a and downregulated Bcl-2. Following miR-34a/p53 expression, cleaved caspase-3 and caspase-8 were activated and apoptosis was induced via the mitochondrial pathway.

XCHD inhibits C6 cell growth which was influenced by the p53/caspase pathway. Thus, the results of this study suggest XCHD could be useful in the inhibition of C6 cells and protecting the glioma.

## Data Availability

The data are presented as mean ± standard deviation (SD); each data was repeated five times.
